# Supplementary material for: The interactome of intact mitochondria by cross-linking mass spectrometry provides evidence for coexisting respiratory supercomplexes
Source: Mol Cell Proteomics. 2017 Dec 8;17(2):216–32. doi: 10.1074/mcp.RA117.000470 (PMC5795388; doi:10.1074/mcp.RA117.000470)
Supplement: Supplemental Data [file supp_RA117.000470_133922_0_supp_23978_fzffwf.zip › spectra_annotation/mito_DR_spectra_annotation/185-1-3-1-4-1.pdf]

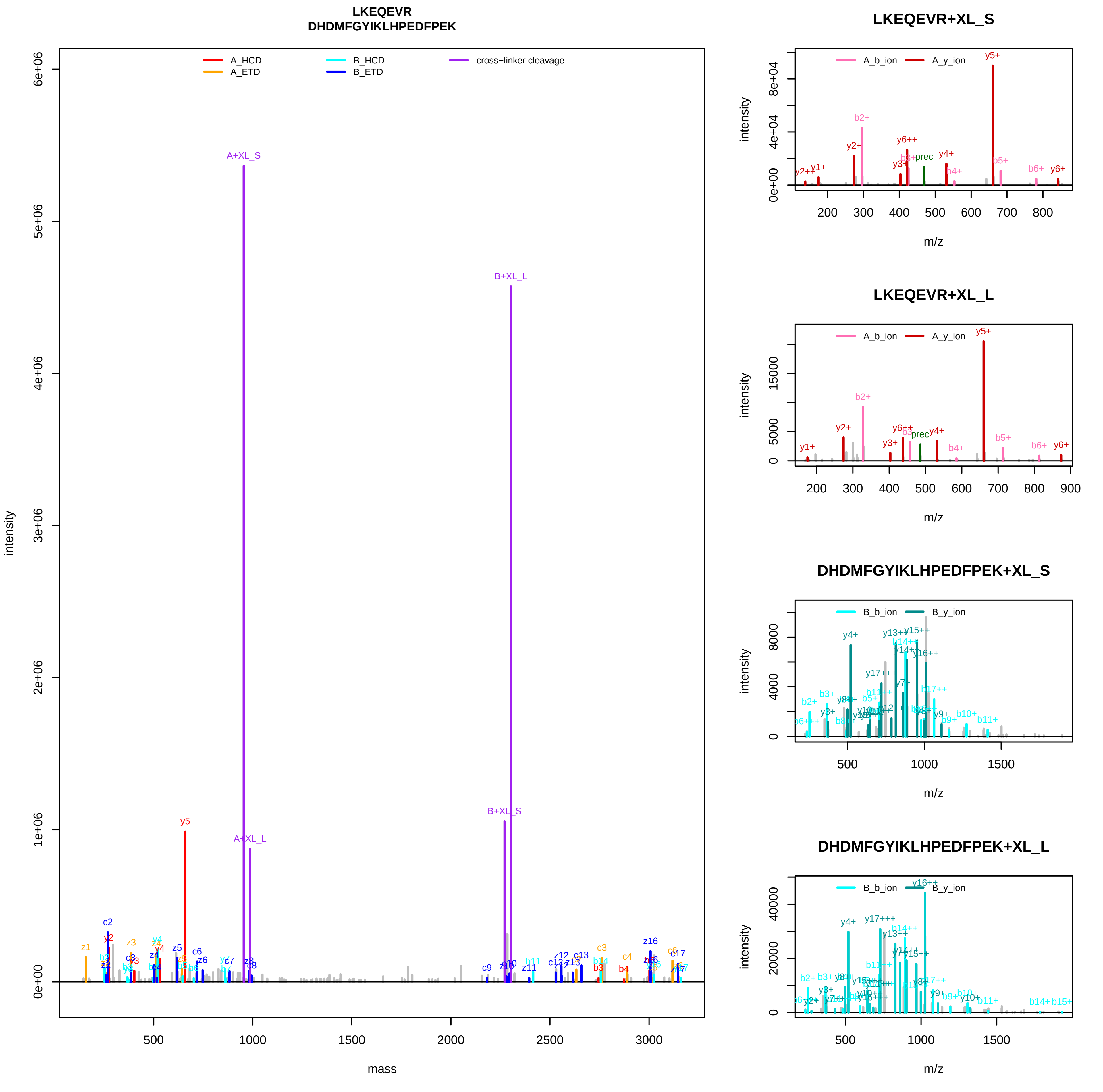

LKEQEVR
DHDMFGYIKLHPEDFPEK
A_HCD
A_ETD
B_HCD
B_ETD
cross−linker cleavage
A+XL_S
B+XL_L
B+XL_S
A+XL_L
y5
c2
z1
y2
z3
y4
z5
c6
z6
c7
c8
c9
z10
b11
z11
c12
z12
z13
c13
c3
b14
c4
b3
b4
z16
c17
intensity
mass
0e+00
1e+06
2e+06
3e+06
4e+06
5e+06
6e+06
500
1000
1500
2000
2500
3000
LKEQEVR+XL_S
A_b_ion
A_y_ion
y5+
b2+
y2+
y6++
y4+
prec
y3+
y1+
y2++
b5+
b6+
y6+
b4+
m/z
0e+00
4e+04
8e+04
200
300
400
500
600
700
800
LKEQEVR+XL_L
0
5000
15000
900
DHDMFGYIKLHPEDFPEK+XL_S
B_b_ion
B_y_ion
y4+
y13++
y15++
y14++
y16++
y17+++
b2+
b3+
y3+
y8++
y9+
b10+
b9+
b11+
0
4000
8000
DHDMFGYIKLHPEDFPEK+XL_L
y16++
y17+++
b14++
y13++
b14+
b15+
20000
40000
